# Is the Fatty Acids Profile in Blood a Good Predictor of Liver Changes? Correlation of Fatty Acids Profile with Fatty Acids Content in the Liver

**DOI:** 10.3390/diagnostics9040197

**Published:** 2019-11-19

**Authors:** Dominika Maciejewska, Joanna Palma, Karolina Dec, Karolina Skonieczna-Żydecka, Izabela Gutowska, Małgorzata Szczuko, Karolina Jakubczyk, Ewa Stachowska

**Affiliations:** 1 Department of Human Nutrition and Metabolomics, Pomeranian Medical University in Szczecin, 70-204 Szczecin, Poland; palma.01.01@gmail.com (J.P.); karolina_dec@wp.pl (K.D.); karzyd@pum.edu.pl (K.S.-Ż.); malgorzata.szczuko@pum.edu.pl (M.S.); jakubczyk.kar@gmail.com (K.J.); ewastachowska.pum@gmail.com (E.S.); 2 Department of Medical Chemistry, Pomeranian Medical University in Szczecin, 70-204 Szczecin, Poland; izagut@poczta.onet.pl

**Keywords:** fatty acid, NAFLD, NASH, lipid marker, non-invasive marker of NAFLD

## Abstract

Background: Existing data show a correlation between the profile of fatty acids, liver, and blood. Therefore, the aim of our study was to investigate the correlation between the fatty acids profile in blood pallets and the liver. Methods: The experiment was performed on 60 eight-week-old male Sprague-Dawley rats. The study group (*n* = 30, 5 groups, 6 rats each) received a cholesterol diet; the control group (*n* = 30, 5 groups, 6 rats each) received standard food for laboratory rats. The rats from both the study and control groups were sacrificed after 2, 4, 8, 12, and 16 weeks of dietary exposure. The fatty acids profile was measured using gas chromatography (GC). Results: In both the control and study group, the highest correlations were observed in palmitoleic acid (RHO = 0.68), heptadecanoic acid (RHO = 0.65), vaccenic acid (RHO = 0.72), eicosapentaenoic acid (RHO = 0.68), docosapentaenoic acid (RHO = 0.77), and docosahexaenoic (RHO = 0.77). Among liver indexes, the highest correlations were desaturase-18 (0.61). Conclusions: Fatty acids profile is a sensitive marker of the development of potentially pathological changes in the liver. The potential markers of fatty liver are: oleic acid, vaccenic acid, EPA, DHA, docosapentaenoic acid, and desaturase index (SCD-18 index).

## 1. Introduction

Lipids are macronutrients that are essential for energy storage, signaling, and membrane structures. They play important roles in many molecular processes due to the wide variety in their structural and physiochemical properties. Lipids play a major function in many diet-related disorders such as obesity, type-II diabetes, and non-alcoholic fatty liver disease (NAFLD). Currently, researchers are searching for non-invasive lipid biomarkers of the metabolic symptoms of many metabolic diseases [1,2]. 

NAFLD is a complex multifactor disorder that affects people around the world. It ranges from simple steatosis, with different degrees of non-alcoholic steatohepatitis (NASH) or fibrosis, to cirrhosis. The disease is closely connected with the metabolism disorders of fatty acids. The pathomechanism includes an increased concentration of free fatty acids (FFA) in blood, an increase in the biosynthesis of fatty acids in the liver [3], as well as disorders of the β-oxidation process [4,5]. NAFLD development is closely associated with the uptake of fatty acids and triglycerides (TGs) from circulation and TG-VLDL secretion from the liver [6]. The only reference method for the evaluation of fatty liver degree is histopathological testing via a liver biopsy. However, because the test is invasive, a complications exists. Thus, to diagnose NAFLD, ultrasonography is often performed combined with a panel of biochemical blood parameters. Unfortunately, this method does not provide a conclusive diagnosis, especially in the case of differentiating between NAFLD and NASH [7]. Excessive change in lipid pathways is also responsible for the progression from simple steatosis to NASH; therefore, liver and blood lipidomic signatures are good indicators of NAFLD progression [2,8]. The profile of fatty acids present in human blood is the result of lipids supplied with diet, lipolytic activity of adipose tissue, and fatty acid biosynthesis [9]. Studies have shown that lipids in plasma/serum are sensitive indicators of short-term changes connected with daily food intake, but erythrocytes/platelets can reflect long-term changes [10]. Therefore, the evaluation of metabolic changes should be estimated according to erythrocyte/platelet analysis [11]. Because the liver is the centre of lipid changes, fatty acids profiling should reflect the pathological changes within this organ. No data have yet reported a correlation between the profile of fatty acids, liver, and blood. Therefore, the aim of our study was to investigate the correlation between the fatty acids profile of blood pallets (containing erythrocytes and platelets) and the liver.

## 2. Materials and Methods

### 2.1. Animals

In the experiment, we used 60 eight-week-old make Sprague-Dawley rats that were randomly assigned into study and control groups by a university staff member who was unrelated to the experiment. The animals had an unlimited supply of food and water and remained in a room with a controlled temperature and a 12-hour light/dark cycle. The study group, which consisted of 30 rats in five groups with six rats each, was supplied with a diet described by Xu et al. [12] that was high in fat and cholesterol. The control group, which consisted of 30 rats in five groups with six rats each, was provided with Rodent Lab Chow (PURINA, Warsaw, Poland). The animals from both groups were sacrificed after 2, 4, 8, 12, and 16 weeks of exposure to the diet using an intraperitoneal (i.p.) ketamine injection. At each of the presented time points, 12 rats were sacrificed, six from each group. In the next stage, blood was collected from the animals’ hearts and they were bled by means of cardiac puncture. The Local Ethical Committee on Animal Testing in Poznań (Poland) approved the experiment (approval No. 76/2016, 16 December 2016). During the entire time of the experiment, no rat died in either of the two groups.

### 2.2. NAFLD Evaluation

The livers were immediately taken for histological examination, fixed in 4% buffered formalin solution, embedded in paraffin and cut into 4-µm sections. For the morphological analysis (Leica DM5000B), serial sections of livers were stained with hematoxylin and eosin (H&E). Hepatic fibrosis was assessed using Mallory trichrome methods (Bio-Optica). Ten light microscopic fields were viewed on each section and scored for the severity of hepatic steatosis and fibrosis [12]. For hepatic steatosis, the following criteria were used: grade 0, no fat; grade 1, steatosis occupying less than 33% of the hepatic parenchyma; grade 2, steatosis occupying in 34–66% of the hepatic parenchyma; and grade 3, more than 66% of the hepatic parenchyma. The following criteria were used to evaluate the staging of hepatic fibrosis: 0, none; 1, mild, zone 3, perisinusoidal; 2, moderate, zone 3, perisinusoidal; 3, portal/periportal; and 4, bridging fibrosis [13]. During the period of 16 weeks, the rats developed different stages of NAFLD, from simple steatosis by four weeks to inflammation with fibrosis by 16 weeks. Table 1 shows the histological evaluation of NAFLD.

### 2.3. Statistical Analysis

The statistical analysis was performed using Statistica 12.0 software. To estimate the correlation, a Spearman correlation test was used (the distribution deviated from normal). The rank correlation coefficient (RHO) was used to determine the strength of the correlation: 0.0–1.9, very weak; 0.2–0.39, weak; 0.4–0.59, moderate; 0.6–0.79, strong; and 0.8–1.0, very strong (*p* < 0.05 was considered statistically important). For the results that were not statistically significant, the abbreviation NS (not significant) was used. 

### 2.4. Fatty Acid Evaluation

Fatty acids were evaluated from blood and liver tissue. We collected 5 mL of blood in vials with heparin and centrifuged (1850× *g*, 4 °C, 10 min) to obtain plasma and blood cells. We then analyzed 50 mg of liver tissue collected from the middle lobe. Then, 0.5 mL of blood cells/50 mg of liver tissue was saponified with 1 mL of 2 M KOH in methanol (70 °C for 20 min) and methylated with 2 mL of 14% boron trifluoride methanolic solution (70 °C for 20 min). For phase separation, 10 mL of saturated sodium chloride and 2 mL of hexane were added. We used 1 mL of the unpolar (hexane) phase for gas chromatography (GC) [14]. An Agilent Technologies 7890A system was used to perform GC with a Supelcowax 10 capillary column (15 m × 0.10 mm, 0.10 μm; Supelco). The initial temperature was 40 °C (0.5 min), which was increased at a rate of 25 °C/min up to 195 °C, 30 °C/min to 205 °C for 0 min, and 80 °C/min to 250 °C for 0.5 min, then maintained for an additional period. The total analysis time was 16.16 min, and the gas flow rate was 1 mL/min, with hydrogen as the carrier gas. Heneicosanoic acid was used as the internal standard.

### 2.5. Stearoyl-CoA Desaturase (SCD) Index and De Novo Lipogenesis (DNL) Index

SCD indexes were calculated using the product-substrate ratio, with SCD-18 oleic acid/stearic acid and SCD-16 = palmitoleic acid/palmitic acid [15,16]. The de novo lipogenesis index (DNL) is calculated according to the following formula: DNL = palmitic acid/linoleic acid [17,18].

## 3. Results

### 3.1. Correlation of Fatty Acids between Liver and Blood Cells in the Control Group

In the control group, the highest correlations were observed in palmitoleic acid, docosahexaenoic acid (DHA), vaccenic acid, and docosapentaenoic acid (Table 2). Among liver indexes, the highest correlation was found in SCD-18 (Table 2). All correlations were of moderate strength.

### 3.2. Correlation of Fatty Acids between Liver and Blood Cells in the Study Group

In the study group, the highest correlations were observed in vaccenic acid and docosahexaenoic acid (Table 3). Among liver indexes, the highest correlation was found in SCD-18 (Table 3). All correlations had moderate strength.

### 3.3. Correlation of Fatty Acids between Liver and Blood Cells in both the Control and Study Groups

In both the control and study groups, the highest correlations were observed in palmitoleic acid, heptadecanoic acid, vaccenic acid, EPA, docosapentaenoic acid, and DHA (Table 4). Among liver indexes, the highest correlations were found in SCD-18 (Table 4). All correlations of fatty acids were strong. 

## 4. Discussion

NAFLD is characterized by significant disorder of lipid metabolism. This condition includes increased endogenic synthesis of saturated fatty acids in the liver as well as the accumulation of lipids provided with the diet. Being the center of energetic changes in the organism, the liver most influences the maintenance of glucose-lipid homeostasis [19]. Therefore, the profile of fatty acids may reflect pathological changes in the organ.

To the best of our knowledge, no data have reported a correlation between a particular fatty acid in the blood and the liver. However, some data have revealed that NAFLD is associated with significant changes in fatty acids profiles in blood. Safaei et al. showed that NAFLD is associated with a significant increase in the oleic acid (C18:1n9) to stearic acid (C18:0) ratio, as well as the palmitoleic (C16:1) to palmitic acid (16:0) ratio. NASH patients were characterized by a decreasing ratio of DHA (C22:6) to docosapentaenoic acid (C22:5) [20]. Our previous research also revealed similar results. We found changes in the fatty acids profiles in patients’ serum, with reductions in liver steatosis by one or two degrees. The reduction in liver steatosis by one degree caused a significant increase in the *n*-3 family levels: EPA (C20:5, *p* < 0.055), docosapentaenoic acid (C22:5, *p* < 0.05), and DHA (*p* < 0.05). A reduction in liver steatosis by two degrees caused a significant decrease in serum palmitoleic acid [21]. The next study conducted by our research team was based on fatty acid erythrocytes membrane profiles and showed that steatosis reduction is related to a significant reduction in stearic (C18:0), palmitoleic, and oleic acids, which tended to increase in polyunsaturated fatty acids (PUFAs) including eicosapentaenoic (*p* < 0.055) and docosahexaenoic acids (*p* < 0.55) [22]. Changes in monounsaturated fatty acids (MUFAs) were also visible in children with NAFLD. Lu et al. conducted a study on 41 Chinese pediatric patients aged 4 to 17, and serum palmitoleic acid concentrations were higher in the mild and severe groups of NAFLD compared with the control [23]. 

Despite the fatty acids profile changes in the blood, some studies have confirmed differences in liver tissue. Puri et al. examined the fatty acids profile in the liver tissues of 18 NAFLD patients (nine with simple steatosis and nine with NASH) and nine healthy controls. NAFLD patients revealed a trend of increased MUFA, specifically oleic acid, in TGs for both simple steatosis and NASH. NASH patients showed lower levels of EPA and DHA [24]. MUFA changes reflect de novo lipogenesis pathways. Stearoyl-CoA desaturase (SCD) plays a major role in the last step of fatty acid biosynthesis, converting saturated fatty acids to MUFAs. SCD catalyzes the synthesis of palmitoleic acid (SCD-16) and oleic acid (SCD-18) by introducing a double bond between the 9th and 10th carbons. Increased SCD activity and a significant correlation between SCD and the severity of steatosis were found in NAFLD subjects [25]. Therefore, the ratio of SCD product-to-precursor was used to evaluate enzyme activity in hepatocytes [26]. However, assessing whether the blood SCD index corresponds to liver SCD activity was difficult.

In this study, we revealed significant and strong correlations between particular fatty acids in blood and the liver. Heptadecanoic acid, oleic acid, vaccenic acid, DHA, and docosapentaenoic acid most reflected changes in the liver (Table 2, Table 3 and Table 4). Oleic and vaccenic acids produced from stearic acid were the most important MUFA. Previous research has revealed that severity of steatosis is related to MUFA changes. In both the control and study groups, the levels of correlation was high (RHO = 0.68 for oleic acid and RHO = 0.72 for vaccenic acid). Separately, in the study and control groups, the RHO levels were lower, which could be caused by smaller amounts of samples in one group (*n* = 36). However, that previous research showed that oleic and vaccenic acids are good predictors of liver changes. The positive correlation between blood and liver confirms that the production of MUFA is increased during the steatosis process, and that it reflects changes in both the blood and the liver. The SCD-18 index of activity also revealed a strong positive correlation (RHO = 0.61 in both groups) with NAFLD progression. This index can reflect NAFLD pathogenesis, which includes increased de novo lipogenesis and TG formation [27,28]. Other important factors that reflect changes in the liver are omega-3 fatty acids, particularly DHA, as well as its precursor docosapentaenoic acid. This observation is especially valuable, as maintaining a proper level of omega-3 fatty acid in NAFLD is essential during treatment [29,30]. Omega-3 fatty acids and their derivatives have strong anti-inflammatory properties that help reduce the metabolic effect of oxidative stress in the liver and other peripheral tissues [31]. The blood levels of EPA, DHA, and docosapentaenoic acid seems to be sensitive to liver changes. The correlations between the liver and the blood of omega-3 fatty acids were high: RHO = 0.77 for DHA and docosapentaenoic acid, and RHO = 0.68 for EPA in both groups.

## 5. Conclusions

The fatty acids profile is a sensitive marker of the development of potentially pathological changes in the liver. Our study revealed a strong positive correlation between fatty acids in blood and the liver, which indicates future applications in fatty acid blood assessment. The potential markers of fatty liver are: MUFA oleic and vaccenic acids, as well as the SCD-18 index and omega-3 fatty acids, including mostly EPA, DHA, and docosapentaenoic acid.

## 6. Limitations

According to recommendation of the Local Ethical Committee on Animal Testing, there were six animal subgroups. This amount of animals is sufficient for performing the appropriate tests, but the results could be improved in a larger group. The study should be repeated in a larger group of animals, especially if human studies can be difficult to conduct (mainly due to liver biopsy in healthy patients).

## Figures and Tables

**Table 1:** Histological evaluation of NAFLD.

**Group**	**Week**	***n***	**Histological Grades of Steatosis**			
			**0**	**1**	**2**	**3**
Control	2–16	36	100%	1	2	3
Study	2	6	100%	0	0	0
	4	6	75%	15%	0	0
	8	6	0	78.30%	21.70%	0
	12	6	0	0	63.33%	36.67%
	16	6	0	0	43.33%	56.67%
**Group**	**Week**	***n***	**Histological Grades of Inflammation**			
			**0**	**1**	**2**	**3**
Control	2–16	36	100%	1	2	3
Study	2	6	100%	0	0	0
	4	6	80%	20%	0	0
	8	6	68.33%	30.00%	1.67%	0
	12	6	63.33%	30%	6.67%	0.00%
	16	6	46.66%	41.67%	11.67%	0.00%
**Group**	**Week**	***n***	**Histological Grades of Fibrosis**			
			**0**	**1**	**2**	**3**
Control	2–16	36	100%	1	2	3
Study	2	6	100%	0%	0%	0%
	4	6	100%	0%	0%	0%
	8	6	100%	0%	0%	0%
	12	6	16.7%	66.6%	16.70%	0%
	16	6	16.7%	50.00%	33.30%	0%

**Table 2:** Correlation of fatty acids between liver and blood cells in the control group.

Fatty Acid, Control	RHO	*p-*Value
C14:0 Myristic acid	0.05	NS
C14:1 Myristolenic acid	0.04	NS
C15:0 Pentadecanoic acid	0.32	<0.01
C15:1 Pentadecenoic acid	0.12	NS
C16:0 Palmitic acid	0.24	NS
C16:1 Palmitoleic acid	0.55	<0.01
C17:0 Heptadecanoic acid	0.34	<0.01
C17:1 Heptadecenoic acid	0.15	NS
C18:0 Stearic acid	0.13	NS
C18:1n9 Oleic acid	0.45	<0.05
C18:1n7 Vaccenic acid	0.53	<0.01
C18:2n6 Linoleic acid	0.29	<0.05
C18:3n6 γ-linoleic acid	(−)0.24	NS
C18:3n3 Linolenic acid	(−)0.22	NS
C18:4 Stearidonic acid	(−)0.24	NS
C20:0 Arachidic acid	0.26	<0.05
C20:2 cis-11-eicodienoic acid	(−)0.11	NS
C20:3n6 eicosatrienoic acid	0.14	NS
C20:4n6 Arachidonic acid	0.28	<0.05
C20:5n3 Eicosapentaenoic acid (EPA)	0.36	<0.01
C22:0 Behenic acid	0.07	NS
C22:2 Docodienoic acid	0.25	NS
C23:0 Tricosanoic acid	0.07	NS
C22:4n6 Docosatetraenoic acid	0.22	NS
C22:5w3 Docosapentaenoic acid	0.52	<0.01
C24:0 Lignoceric acid	0.041	NS
C22:6n3 Docosahexaenoic acid (DHA)	0.54	<0.01
C24:1 Nervonic acid	0.11	NS
**Parameter**		
SCD-16	0.42	<0.01
SCD-18	0.55	<0.01
DNL	0.11	NS

**Table 3:** Correlation of fatty acids between liver and blood cells in the study group.

Fatty Acid, NAFLD Groups	RHO	*p-*Value
C14:0 Myristic acid	0.08	NS
C14:1 Myristolenic acid	0.13	NS
C15:0 Pentadecanoic acid	0.31	0.01
C15:1 Pentadecenoic acid	0.09	NS
C16:0 Palmitic acid	0.21	NS
C16:1 Palmitoleic acid	0.43	NS
C17:0 Heptadecanoic acid	0.39	<0.05
C17:1 Heptadecenoic acid	0.11	NS
C18:0 Stearic acid	0.22	NS
C18:1n9 Oleic acid	0.42	<0.05
C18:1n7 Vaccenic acid	0.47	<0.01
C18:2n6 Linoleic acid	0.17	<0.05
C18:3n6 γ-linoleic acid	(−)0.2	NS
C18:3n3 Linolenic acid	(−)0.26	NS
C18:4 Stearidonic acid	(−)0.03	NS
C20:0 Arachidic acid	0.21	NS
C20:2 Eicodienoic acid	(−)0.05	NS
C20:3n6 Eicosatrienoic acid	0.18	NS
C20:4n6 Arachidonic acid	0.28	NS
C20:5n3 Eicosapentaenoic acid (EPA)	0.33	<0.05
C22:0 Behenic acid	0.04	NS
C22:2 Docodienoic acid	0.15	NS
C23:0 Tricosanoic acid	0.10	NS
C22:4n6 Docosatetraenoic acid	0.41	<0.05
C22:5w3 Docosapentaenoic acid	0.31	<0.05
C24:0 Lignoceric acid	0.22	NS
C22:6n3 Docosahexaenoic acid (DHA)	0.53	<0.01
C24:1 Nervonic acid	0.013	NS
**Parameter**		
SCD-16	0.22	<0.05
SCD-18	0.51	<0.05
DNL	0.17	NS

**Table 4:** Correlation of fatty acids between liver and blood among all groups.

Fatty Acid (Both Groups)	RHO	*p-*Value
C14:0 Myristic acid	0.09	NS
C14:1 Myristolenic acid	0.03	NS
C15:0 Pentadecanoic acid	0.63	<0.01
C15:1 Pentadecenoic acid	0.06	NS
C16:0 Palmitic acid	0.51	<0.01
C16:1 Palmitoleic acid	0.64	<0.01
C17:0 Heptadecanoic acid	0.65	<0.01
C17:1 Heptadecenoic acid	0.16	NS
C18:0 Stearic acid	0.21	NS
C18:1n9Oleic acid	0.68	<0.05
C18:1n7 Vaccenic acid	0.72	<0.01
C18:2n6 Linoleic acid	0.39	<0.05
C18:3n6 γ-linoleic acid	(−)0.3	<0.05
C18:3n3 Linolenic acid	(−)0.27	NS
C18:4 Stearidonic acid	(−)0.01	NS
C20:0 Arachidic acid	0.39	<0.05
C20:2 Eicodienoic acid	(−)0.17	NS
C20:3n6 Eicosatrienoic acid	0.26	<0.05
C20:4n6 Arachidonic acid	0.59	<0.01
C20:5n3 Eicosapentaenoic acid (EPA)	0.68	<0.01
C22:0 Behenic acid	0.14	NS
C22:2 Docodienoic acid	0.19	NS
C23:0 Tricosanoic acid	0.15	NS
C22:4n6 Docosatetraenoic acid	0.35	NS
C22:5w3 Docosapentaenoic acid	0.77	<0.01
C24:0 Lignoceric acid	0.14	NS
C22:6n3 Docosahexaenoic acid (DHA)	0.77	<0.01
C24:1 Nervonic acid	0.07	NS
**Parameter**		
SCD-16	0.42	<0.01
SCD-18	0.61	<0.01
DNL	0.19	NS
